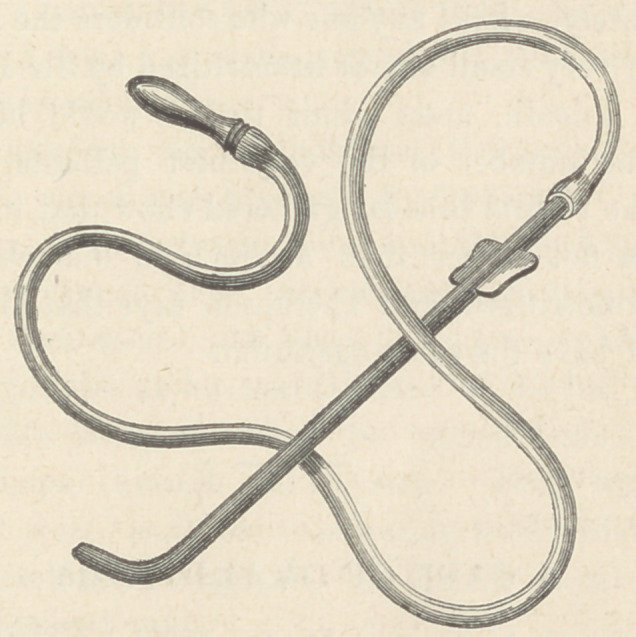# Cases of Lithotrity ; an Instrument for Finding Small Remaining Fragments of Stone by Auscultation

**Published:** 1878-06

**Authors:** Edmund Andrews


### CASES OF LITHOTRITY; AN INSTRUMENT FOR FINDING SMALL REMAINING FRAGMENTS OF STONE BY AUSCULTATION.

By Edmund Andrews, A. M., M. D.

There is no denying the fact, that we of the western continent have grossly neglected the operation of lithotrity, and I confess to my full share of the sin. We have gone on cutting patients by scores and hundreds, when a large portion of them would have been more safely treated by the operation of crushing.

In children, cutting is, to be sure, very safe, and crushing nearly impracticable, but in adults the facts are as follows:

Mortality of lithotomy in patients over 20 years of age.

| | Cases. | Deaths. | Per cent. mortality. |
|---|---|---|---|
| Sir Henry Thompson's table | 723 | 150 | 21 |
| Keith's table, *Brit. Med. Jour.* March 20, 1868 | 1312 | 330 | 25 |
| | 2035 | 480 | 23 |

Thus the best authorities in the world give us a mortality of over 23 per cent. for adult lithotomy, or nearly one death in four.

Now contrast this with the results of lithotrity. The following table shows the results of the operation on both continents:

LITHOTRITY.

| Authorities. | Cases. | Deaths. |
|---|---|---|
| Transactions of N. Y. State Med. Society | 49 | 9 |
| Bigelow, of Boston | 6 | 1 |
| Andrews, of Chicago | 6 | 0 |
| Eve, of Nashville | 4 | 0 |
| Drs. Curtis and Porter, quoted by Bigelow | 2 | 0 |
| Report Boston City Hospital | 1 | 0 |
| Report Pennsylvania Hospital | 14 | 2 |
| Brodie, of England | 115 | 9 |
| Sir Henry Thompson, of England | 422 | 32 |
| Fergusson, of England | 109 | 12 |
| Keith, of Scotland | 116 | 7 |
| Crichton | 122 | 8 |
| Statistics des Hôp. de Paris, 1861-2-3 | 56 | 9 |
| Civiale of Paris | 591 | 14 |
| K. K. allg. Krankenhaus, Wien | 42 | 16 |
| Lücke, of Berne | 2 | 0 |
| Dr. Kerr, of the Missionary Hosp. Canton, China | 30 | 3 |
| | 1687 | 122 |

Mortality 7 per cent.

As stated above, this shows that lithotrity in adults is very much safer than lithotomy. A glance at the table also shows how American surgeons have neglected the operation. Paul F. Eve's four cases are given in the same paper with a hundred cases in which he performed lithotomy. The Boston City hospital reports only one case, the State Medical Society of New York, 49 cases, and the Pennsylvania hospital only 14 cases, while the principal British surgeons tabulate them by hundreds, and even a distant missionary physician in China sends thirty cases.

I confess to my share of blame in operating heretofore almost altogether by cutting, but the increasing success of lithotrity in

Europe renders it impossible for American surgeons to longer ignore its claims in a suitable selection of cases. I have there-therefore crushed the stone of late in eight different cases, and thus far without a death. Two of the cases, however are unfinished, so that I cannot include them in the table.

Case I. Adult male. Stone in the bladder, accreted around a roll of chewing gum, which the patient introduced a year ago. Urethra was contracted too small for lithotrite, I therefore kept up a course of dilatation with bougies for two or three weeks before operating. The calculus was then attacked, and crushed at ten sittings in the course of nineteen days. Several subsequent examinations having failed to discover any remnant of the stone or of the gum, the patient was discharged, and is not known to have had any relapse. No dangerous symptoms occurred during the treatment.

Case II. Patient was caught under a mass of falling rock, and received an injury of the spinal cord, which paralyzed the inferior extremities almost completely. About a year later he showed signs of a calculus in the bladder. Eleven months later he came under my care. The lower extremities were still paralyzed, and the bladder with them, rendering a frequent use of the catheter necessary. Exploration showed a stone with a diameter of about an inch. The bladder was frequently affected with some hemorrhage. I gave him five days preparation, by giving freely quinine and bromide of potassium. On the sixth day I crushed the stone. Some cystitis was provoked, but rapidly subsided, and I repeated the crushing on the 8th day. Then followed four days of diarrhœa, which being subdued, I crushed again, on the fifteenth day. On the seventeenth day some pieces became impacted in the urethra and were removed, with great relief. Additional sessions were had on the 19th, 21st, 22d, 25th, 32d and 36th days. After the latter session there was a brisk temporary hemorrhage of the bladder, filling that viscus with clots, which could not be expelled by the patient. They were drawn out by a strong syringe attached to one of the large catheter-like tubes of the wash-bottle. There was no serious inflammation, and on the 44th day the eleventh and last session was had. Several subsequent examinations showed that no more stone was present, and the patient was sent home. The stone was phosphatic but rather hard. This was a difficult and tedious case, but at no part of the treatment did any alarming symptoms arise.

Case III. This case is remarkable for being executed in defiance of the rule that the patient should observe the recumbent position after each operation. He was a healthy man, about thirty years of age, and lived in a suburban town twelve miles distant from my office. The stone measured a little less than three-quarters of an inch in diameter and was rather soft. The patient declared that there was no arrangement possible in his circumstances, but to come to my office for his operations, and then to return home by rail the same evening. Seeing that the rules could not be enforced, I determined to try him cautiously as he proposed. His cystic trouble was of six years' duration, but the symptoms were quite mild. I therefore very cautiously seized the stone and crushed it, kept him on the lounge an hour or two, and then sent him home to his family physician. Sixteen days later I repeated the operation, and two days afterwards made the final crushing, three sessions in all. Subsequent soundings showed no more stone, and he was discharged cured. This case turned out well, but it would certainly be a rash plan to allow ordinary parties to risk themselves by a twelve-mile ride after each session.

Case IV. Patient aged 50 years; bladder very sensitive; stone rather more than an inch in diameter, pretty hard and of two years growth. I proceeded at first with great caution, allowing plenty of time between the sessions. Ten sessions were had scattered through two months time. There was quite a tendency to urethral chills after the sessions, which I restrained by large doses of quinine. However, he grew much more tolerant of instruments as time progressed, and was discharged cured.

Case V. Patient 68 years of age, and greatly troubled with his disease. Twenty years before, he had been lithotomized, and had 17 small stones removed at once.

The present stone was half an inch in diameter, very soft and quite difficult to find and seize. I crushed it at three sessions, but had several searches in which I failed to grasp it, and dared not continue the manipulations long, on account of the constant tendency to cystitis, urethral chills and hæmorrhage. Once the bladder filled with clots obliging me to empty it by the suction of a strong syringe through a No. 12 catheter.

Quinine was used freely to restrain the chills and a steady use of benzoic acid by the mouth greatly relieved the cystic irritation. The latter remedy was used in consequence of a suggestion of Dr. Lucius Clark, of Rockford. The patient was discharged cured.

Case VI. Patient aged about 48 years, and in medium condition. The stone was nearly three quarters of an inch in diameter, and excessively hard, being composed chiefly of oxalate of lime.

I crushed four or five times, besides making a few fruitless searches, during 22 days. No severe symptoms occured. The patient was kept on cinchona preparations copiously, and was discharged cured.

Case VII. Patient aged 54. Case unfinished.

Case VIII. Patient aged 64. Feeble. Case unfinished.

Prof. H. Bigelow, of Boston, published in the last number of the *American Journal of the Medical Sciences*, an article on the performance of lithotrity by a single operation. He gives five cases of his own and one of another surgeon, operated on at a single sitting. One of the six died. The operations lasted on the average nearly an hour and a half and the patients were under ether. Dr. Bigelow thinks it is reasonably safe to rid the patient completely of the stone at once, and thus save the irritation of the remaining fragments and the loss of time involved in the slow method. His mortality of one patient in six is a bad result, so far as it goes, but the number being so small one cannot conclude with certainty that more experience would prove equally dangerous. My own opinion however, is that the safety of lithotrity lies in the fact that the operation can be done a little at a time. In my experience prolonged working in the bladder with the lithotrite is decidedly more irritating than short sessions. In stones of average size, and bladders of corresponding degree of inflammation, one of these hour and a half sessions will probably be as dangerous as lithotomy, but in very small stones contained in bladders of little irritability, I think the plan of finishing the work at once will be found safe and judicious.

Prof. Bigelow has made some useful improvements in the tube and wash bottle. He has also studied the action of instruments in dead bodies by inserting them into the bladders, and then injecting them with plaster of paris. The plaster when hardened gave the form of the vesical cavity as modified by the pressure of the instruments at various points. The article is highly instructive, and shows the principles which should guide one in searching for fragments, and how pressure of the instrument at the lower fundus of the bladder makes a funnel-shaped hollow where all fragments tend to fall into the jaws of the lithotrite or the orifice of the wash tube.

One of the difficult points in lithotrity is to know when all the small fragments are removed. To facilitate the discovery of the last small bits, I have devised an auscultating sound, which will convey to the ear the faintest touch of a particle of stone.

This instrument consists of a metallic searcher of the ordinary form, but made hollow. To the outer end is attached a small rubber tube and ear piece, like those used by aural surgeons in listening to the tympanum. This transmits to the ear of the surgeon with great distinctness the sound of very minute particles of stone, and adds greatly to the feeling of security when, on the final search, the patient is discharged as cured.

I cannot but feel that American surgeons, myself included, have been negligent in cutting rather indiscriminately large numbers of patients, when about one half of them could have been treated more safely by lithotrity. However, let no one be so enthusiastic as to suppose that the crushing operation should supersede cutting in all cases. There is perhaps, no more enthusiastic lithotritist living than Sir Henry Thompson, and his matured opinion delivered only a few weeks ago, is that if lithotrity be applied to too large stones—that is to stones much above the size of an almond, the results will not be superior to those of lithotomy in the same class of cases.

The table of lithotrity, given above, shows nominally that the operation kills only one-third as many patients as adult lithotomy; but this, if taken without allowance, would overstate the difference between the operations. The lithotrity patients, on the average, are selected from those having the smallest stones and the soundest bladders, while the lithotomy cases are taken indiscriminately. Still any one who considers the great numbers of men with very small stones lithotritized by Sir Henry Thompson without a death, must admit that it would be impossible to cut the same number of the very best patients without some deaths. The results thus far gathered show that in adults, where the stone is not larger than an inch in diameter, most cases should be lithotritized, and Thompson says that all adult female cases should have the same operation.

## Figures and Tables

**Figure f1:**